# Outcomes identified and prioritised by consumers of Partners in Recovery: a consumer-led study

**DOI:** 10.1186/s12888-017-1498-5

**Published:** 2017-10-06

**Authors:** Shifra Waks, Justin Newton Scanlan, Bridget Berry, Richard Schweizer, Nicola Hancock, Anne Honey

**Affiliations:** The University of Sydney, Faculty of Health Sciences, PO Box 170, Lidcombe, NSW 1825 Australia

**Keywords:** Consumer-led research, Service-user led research, Recovery, Service coordination, Brokerage, Severe and persistent mental illness

## Abstract

**Background:**

Recovery oriented service provisions means focusing on outcomes that are important to consumers themselves rather than to clinicians or services. Partners in Recovery (PIR) is an Australia-wide initiative designed to provide service coordination and brokerage for individuals with severe and persistent mental illness. One PIR service engaged a consumer-led research team to evaluate the service from the perspective of consumers. This consumer-led study was established to explore PIR consumers’ perceptions of outcomes they achieved through their involvement with PIR.

**Methods:**

Data were collected through semi-structured interviews exploring participants’ views about and experiences with PIR. Data analysis occurred simultaneously with data collection using constant comparative analysis.

**Results:**

Twenty consumers participated. They reported experiencing valued outcomes in six domains: *feeling supported*; *feeling more hopeful and positive about the future*; *improved mental clarity, focus and order in life*; *getting out of the house and engaging in positive activity*; *having a better social life*; and *improved physical health*.

**Conclusions:**

Exploring outcomes achieved by PIR consumers, from their own perspective provides a nuanced understanding of the contribution these programs can have in supporting individuals’ recovery. Findings from this study highlight the kinds of outcomes consumers achieve when engaged with service coordination and brokerage services. Findings also suggest that outcome measures used in these types of services should focus on recovery outcomes as well as met and unmet needs.

## Background

It is estimated that approximately 3 % of Australian adults experience “severe and persistent mental illness” [1], characterised by persistent symptoms, significant functional impairment and psychosocial disability. Individuals living with severe and persistent mental illness may have become disconnected from social or family support networks and may require support from a range of agencies across different sectors [2, 3]. However, the mental health service system in Australia has been criticised as fragmented and difficult to access and navigate, especially for those who are most vulnerable [4]. Partners in Recovery (PIR) is an Australia-wide initiative designed to address this issue. PIR aims to better support people living with severe and persistent mental illness by providing a coordinated response to address their complex, diverse range of needs [2, 5].

In the PIR model, a Support Facilitator (SF) works together with a consumer to identify and coordinate their care needs. The SF role is “dominated by efforts to seek out, establish and maintain connections of use in addressing consumers’ needs” ([4], p. 32). As the name suggests, the overarching aim of PIR is to support individuals in their journeys of recovery from mental illness [6]. Within the Australian and international mental health context, recovery-oriented practice is now considered the standard framework guiding mental health services [7]. A recovery-oriented approach is based on developing an understanding of consumers’ perspectives and upholds that consumers are the experts in their own care and recovery [8, 9].

The services PIR provide are typically designed around consumers’ self-identified needs, captured through the Camberwell Assessment of Need – Short Appraisal Scale (CANSAS) [10]. The CANSAS is also a primary outcome measure used across all PIR services [2]. However, many PIR services have expressed concern about the CANSAS as an outcome measure, as it is not well aligned with recovery principles [11]. To be truly recovery-oriented, the evaluation of outcomes also needs to focus on consumers’ own perceptions of services and the outcomes they seek to achieve and that are important to them.

The aim of this paper is, therefore, to explore consumers’ perspectives on the outcomes they achieved through their involvement with PIR.

## Methods

The findings reported in this paper are part of a larger consumer-led evaluation project exploring consumer experiences and outcomes associated with their involvement in one PIR program. A qualitative research approach was used to enable a rich and detailed exploration of participants’ experiences and to collect data on aspects of their experiences that were most important to them [12]. The overall study was approved by the University of Sydney Human Research Ethics Committee (protocol number 2015/510). Procedures used in the study are described according to the consolidated criteria for reporting qualitative research (COREQ) checklist [13].

### Consumer-led research

Recognising the benefits of consumer-led research, the PIR service specified that this evaluation be led by consumer researchers. Consumer-led research is distinct from other forms of consumer involvement in research in that consumer researchers hold ultimate decision making responsibility for all aspects of the research process including: designing the research question, developing the research method, data collection, analysis and report writing. This study was undertaken by three consumer researchers who designed and conducted the research. They were supported by three non-consumer academic researchers, who provided consultation, suggestions and methodological support.

Consumer leadership of the research process has been purported and demonstrated to strengthen both the quality and translation of research and achieves the goal of inclusive research practice “nothing about us without us” [14, 15]. Due to unique knowledge developed through lived experience of mental illness and recovery, consumer researchers focus on different areas and ask different research questions [16]. For this reason, consumer involvement in mental health research, particularly consumer-led research is suggested to deliver research outcomes of greatest importance and impact [16, 17].

### Research team

Interviews were conducted by authors SF (female), BB (female) and RS (male). At the time of the study, they were employed as consumer researchers on the project. In the context of consumer-led research, the primary qualification of the consumer researchers was their lived experience of mental illness and recovery. However, each also had experience in research, with two having engaged in research as part of a higher degree. Training and ongoing support was provided to the consumer researchers by the academic research team who are experienced researchers holding doctoral qualifications. Additionally, the entire research team met together regularly to discuss the research process, and reflect on the emerging themes.

None of the researchers had worked for or received services from PIR; neither had they any existing relationships with the participants. As age, gender and cultural background was diverse amongst both participants and interviewers, these factors are unlikely to have had a systematic effect on data collection overall. From the beginning of the research process, participants were made aware that interviewers had lived experience of mental illness and that the aim of the project was to understand the experiences of PIR clients to support the development of better services for consumers. The researchers all became involved with the study based on core beliefs about the importance of a) understanding consumers’ subjective experiences of mental health services and b) including consumers in research from conceptualisation to dissemination.

### Study design

The assumptions of constructivist grounded theory [18] directed the project and grounded theory techniques were used to develop a preliminary conceptual framework to describe and explain the experiences of consumers. Constructivist grounded theory acknowledges that participants’ stories are developed through dialogue with the interviewer and are interpreted based on researchers’ beliefs, values, previous understandings and experience. This was seen by the research team as consistent with consumer-led research as it implies the importance of research about consumers being developed and interpreted by consumers. Although our analysis represents one interpretation of consumers’ experiences with PIR, its grounding in empirical data, use of a systematic set of grounded theory techniques, and inclusion of multiple researchers, including consumers, in interpretation, provided a rigorous study design.

### Sampling and recruitment

In an earlier stage of the project, a questionnaire was used to gather feedback from current and previous consumers. In this questionnaire, participants were able to consent to being contacted to participate in semi-structured interviews. Interview participants were purposively selected to ensure a spread of demographic characteristics as well as experiences with PIR.

### Data collection

Semi-structured interviews were guided by an interview guide and covered topics such as the general consumer experience of PIR and working with the SF, goals and the goal setting process, recovery and wellness and ways in which PIR differed from other support services. Interviews were held in a private room at the offices of the PIR service, or over the telephone, depending on the participant’s preference. Given that some interviews were held in the offices of the service, care was taken to reassure participants that their responses were completely confidential and individuals would not be identifiable in reports or publications. Interviews were audio recorded and took from 17 min to 1 h 18 min.

Following each interview, the consumer researchers engaged in debriefing to discuss what they had heard, emerging themes, any unforeseen issues or queries and whether any modifications to the interview guide were needed before conducting following interviews.

### Data analysis

Interview recordings were transcribed verbatim. NVivo 11 [18] was used for data management and coding. Analysis occurred simultaneously with data collection following principles from grounded theory [19]. Data were analysed using constant comparative analysis. This involved inductively coding each small unit of data (words, phrases and sentences) and closely comparing data with all other units of data coded, grouping those that expressed a single idea into a single code and comparing against existing codes to determine the need for new codes [20]. Individual codes were compared to each other and grouped into categories in a process of ongoing refinement. The coding process required researchers to set aside previous assumptions and develop codes and categories directly from the data rather than coding data to fit existing theories [20].

Three consumer-researchers and one academic researcher independently coded the first two transcripts and met to discuss and find consensus in the coding and interpretations. As data collection progressed, the coding process was taken on by one consumer researcher (RS) who discussed the data and developing codes in detail with one academic researcher. The emergent codes and categories were discussed throughout the process with other research team members, in particular with the two consumer researchers who conducted the interviews. Interviews proceeded until “data saturation” was achieved, that is, when information from later interviews did not reveal any new categories or themes.

## Results

Twenty individuals completed interviews. Demographic information is summarised in Table 1.

**Table 1:** Demographic data

	Mean (S.D.; range)	
Age	48y (13.7y; 25 to 74y)	
	n	%
Gender		
Female	9	45%
Male	11	55%
Diagnosis		
Major depression	5	25%
Schizophrenia / Schizoaffective disorder/Psychosis	7	35%
Anxiety disorders	5	25%
Post-Traumatic Stress Disorder	2	10%
Bipolar disorder	2	10%
Other	7	35%
Not reported	2	10%
PIR Status		
Current	15	75%
Exited	5	25%

Note: Participants could list more than one diagnosis, so percentages do not add up to 100%

Analyses of interview data identified three linked categories: qualities of a good SF, practical supports that mattered to consumers and outcomes achieved through engagement with PIR. Consumers described how the qualities of a good SF and the practical supports offered by the SF and PIR supported and enabled outcomes to occur in their lives. The focus of this paper is on the third category: outcomes achieved through engagement with PIR.

Although specific outcomes were unique to each individual, they occurred in six domains: *feeling supported*; *feeling more hopeful and positive about the future*; *improved mental clarity, focus and order in life*; *getting out of the house and engaging in positive activity*; *having a better social life*; and *improved physical health*. Descriptions of domains are supported by direct quotes from participants. To protect individuals’ identity, pseudonyms have been used.

### Feeling supported

Participants described the importance of feeling supported. This established a foundation for moving forward with a sense of purpose and helped consumers to feel more socially connected.

This has helped me actually get on with things because I know I’m getting support from other people and I don’t have to sit down and feel paralysed but I can have some help… (Jeff)

It’s really changed my life in so many different ways … I don’t even know them and … they are just so eager to help me, to be there for me. (Angela)

For many participants, this feeling of support included confidence that the SF would stick by them through the ebbs and flows of mental illness.

It's very cyclical for me with bipolar as my condition, so there's plenty of times of wellness, but then when there's times of, you know, illness, then it's good to know that there's a person there to keep supporting you. (Molly)

When you're in a psychotic state, and you're unwell, even when you say you don't want help, you really do… They helped build a lifeline for me through that contact and through that engagement and involvement. (Edward)

Overall, a positive, supportive relationship developed with the SF and this often represented an important “first step” to achieving other outcomes in participants’ recovery journeys.

### Feeling more hopeful and positive about the future

Participants also reported that their participation in the program helped them to gain hope, confidence and motivation. Alongside feeling supported, this sense of hopefulness and positivity allowed participants to take steps towards achieving goals in other areas of their lives.

He just helped kick things off and just made it possible to see some light at the end of the tunnel. Just to be able to work through a process, put something in action and work through it and get something done. It helped me achieve goals that I needed to achieve… (Michael)

They've helped me get more confident. They've helped me just want to continue living. They’ve helped me... just by giving me confidence and encouraging my artwork and my writing and just listening. (Allura)

### Increased mental clarity, focus and order in life

Participants described that the challenges of living with a mental illness and dealing with other life circumstances often resulted in feeling “completely confused” (Michael) or “topsy turvy” (Jeff). Participants described how, through their involvement with PIR, they were able to achieve greater mental clarity, focus and order in their lives. These changes occurred in different ways. Some participants described being supported to see their situations more clearly.

[My SF] helped get a lot of things untangled and just help[ed] me get some focus and… just good support to help me to get some basic day-to-day stuff sorted out… (Michael)

Others discussed gaining greater control and power over the impact of their symptoms, which allowed them to think more clearly and gain strategies to manage their condition. For Allura, this was: “just stopping responding to the voices when they attack… and not feeling compelled to respond.”

For still others, this outcome was about achieving greater order in their lives, for example, through developing practical strategies and routines.

I stick bills and things up there so they’re not all piling up around the place and stuff to remind me of specials at [the supermarket] and the number for housing commission maintenance… so they’re not in a pile somewhere that I’ve got to go search which is stressful. So that’s been useful. (Jeff)

Every time [my SF] came around, it was just this accountability thing that she helped me with the cleaning part of it and that each time I had an appointment with her… My husband says that, in the last year, that there's been a remarkable change in my ability to function in the house. That's good. (Molly)

### Getting out of the house and engaging in positive activity

Many participants reported that when they commenced with PIR, they struggled to find meaningful ways to use their time. For some, the simple act of leaving the house represented a positive change in their lives.

[S]o it’s very much about being able to get out of the house and feeling that’s good, and not just out of the house, but then being able to go and do particular things, a choice of things in a day. (Jeff)

I used to go out once a fortnight and be cooped up behind my computer most of the time… I'm going out more now… At first I used to buy two bus tickets a fortnight, now I buy five. (Allura)

PIR was able to assist participants to get back to doing things they enjoyed and to find new activities that brought meaning into their lives. For Jennifer, this involved being supported to find volunteer work: “I had an interview and they said I can come and do that… I’m quite happy with that…”.

### Having a better social life

Most participants reported having been disconnected from social contacts and needing help to reconnect, maintain and establish new relationships. For many participants, the positive relationship developed with their SF provided a strong sense of improved social connection.

[My SF] would meet with me and take me out… That was really important, because in those early days when I was very reclusive, it was very difficult for me to make that kind of social connection with anybody, so I felt very isolated… those various outings were really critical in my reintegration into society.(Edward)

Importantly however, SFs also facilitated social connections with other people.

[PIR] has made my life better because I was looking for some kind of support group for people with a mental illness. That's why I go to [a community centre]… It's somewhere that I feel I could go when I'm not well as well as when I'm well. (Francis)

[A PIR referral led to a service provider organising] a woman to come up and have a cup of coffee with me for one and a half hours. We had lots to chat about. That's something to look forward to, a highlight of my week. (Patricia)

For one consumer, improving social connections was about building better relationships within the family unit.

…they're helping not just me but my family… The most important thing for me was making sure that the impact that my mental illness has on the family isn't a great impact. That mental health prevails and mental illness is not a consideration when it comes to the kids and their well-being. (Molly)

### Improved physical health

Overcoming challenges associated with their physical wellbeing was important for many participants. Not only did they face challenges associated with medication side effects and physical health effects of mental illness, but many also lived with significant medical comorbidities. These presented multiple challenges for participants. Support provided through PIR allowed a number of participants to access services they required, or to engage more effectively in rehabilitation programs.

I'm starting to age and have physical health problems as well. I was having trouble accessing different services, knowing where to go, and somehow or another, PIR came up as a possibility to help me find some direction. (Rae)

[My SF] is slowly getting back to getting me to go to the pool. Going in the water, and doing walking in there. That's sort of a positive thing… [When I said] "I won't be able to swim," she said, "Remember, you did it last time when you went in." She's there as a safety cushion. (Patricia)

PIR also supported some participants to engage in exercise, such as going for regular walks or accessing a personal trainer.

[My SF] said that, you know, you may be able to benefit from getting involved in some kind of exercises… [The personal trainer] was just incredible in drawing me out and taking me out to the local park and just going through a variety of exercises and the point of that was helping me focus on my physical self, and not just my mental state… [It is] a critical element in a person's well-being, a healthy mind and body. (Edward)

## Discussion

PIR is an innovative model of service delivery, focused on service coordination and brokerage to more effectively address the complex needs of individuals with severe and persistent mental illness. This approach to service delivery is a central feature of many service innovations currently occurring in Australia and around the world e.g., [21, 22]. Therefore, it is of great importance to explore the effectiveness of these services models. To be recovery-oriented, the outcomes that define an effective service need to move beyond outcomes determined by service providers to examine the outcomes valued by consumers themselves. This consumer-led study explored self-identified outcomes achieved by people with severe and persistent mental illness through their involvement with PIR. This builds on the small but important body of literature exploring consumers’ perceptions of the outcomes achieved through their engagement with PIR [23].

Participants reported a range of valued outcomes they had achieved through engagement with PIR. Although outcomes were different for each individual, they fell into six domains: *feeling supported*; *feeling more hopeful and positive about the future*; *improved mental clarity, focus and order in life*; *getting out of the house and engaging in positive activity*; *having a better social life*; and *improved physical health*.

Currently, the CANSAS is the primary outcome measure used by PIR services. It includes 22 items, to which 3 items were added specifically for PIR purposes [24]. The CANSAS requires the SF, through an interview with the consumer, to identify whether there is an unmet need in each of the 25 areas. Some of the outcomes described above are captured, at least in part, by items on the CANSAS. *Getting out of the house and engaging in positive activity* aligns with two items of potential need: ‘daytime activities’ and ‘employment and volunteering’; *having a better social life* is covered by the item entitled ‘company’ and *improved physical health* parallels the item ‘physical health’. The CANSAS item ‘Looking after the home’ reflects one specific aspect of the domain *improved mental clarity, focus and order in life* but the CANSAS fails to capture the overall sense consumers described of reduced confusion and chaos in their lives. The domains of *feeling supported* and *feeling more hopeful and positive about the future* are not captured by the CANSAS, possibly because they are more intrinsic and personal changes that are not well suited to conceptualisation as “unmet needs.”

The CANSAS has been criticised as having a “poor alignment with a recovery approach” [11, p. 50]. While the client-centred nature of the CANSAS is aligned to a recovery-oriented approach, reducing unmet needs and recovery are clearly different things [25]. While outcomes described by participants in this study align somewhat with CANSAS items, they more clearly reflect concepts identified in recovery-based frameworks. Two of the most influential frameworks have been the CHIME framework [26] and the domain-based framework developed by Lloyd and colleagues [27]. The CHIME framework includes five “recovery processes”: *Connectedness*; *Hope and optimism about the future*; *Identity*; *Meaning in life*; and *Empowerment* [26]. Similarly, Lloyd and colleagues [27] identified four domains of recovery: *clinical* (managing the impact of symptoms); *personal* (developing hope for the future and overcoming the trauma associated with experiencing mental illness and regaining a sense of personal responsibility and agency); *functional* (gaining and maintaining socially and personally valuable roles); and *social* (establishing and expanding supportive and satisfying social networks).

This alignment between consumers’ reported outcomes and recovery frameworks suggests that using a measure of recovery may be a useful addition to the CANSAS in monitoring outcomes for PIR as well as other service coordination and brokerage models. Two commonly-used measures of mental health recovery are the Mental Health Recovery Star MHRS: [28] and the Recovery Assessment Scale – Domains and Stages RAS-DS: [29]. Table 2 provides an overview of the alignment between outcomes reported by participants in this study and items included in the CANSAS, MHRS and RAS-DS.

**Table 2:** Comparison of outcome domains from this study and items on various outcome measures

Outcome domain	CANSAS Items	MHRS Items	RAS-DS Items
Feeling supported	n/a	Trust (and hope)	I have people that I can count on Even when I don’t believe in myself, other people do I know that there are mental health services that help me
Feeling more hopeful and positive about the future	n/a	Identity and self-esteem (Trust and) hope	I have the desire to succeed I have goals in life that I want to reach I believe that I can reach my current personal goals Something good will eventually happen I am hopeful about my own future
Improved mental clarity, focus and order in life	Looking after the home	Managing mental health Living skills Responsibilities	I am the person most responsible for my own improvement I know what helps me get better There are things that I can do that help me deal with unwanted symptoms My symptoms interfere less and less with my life
Getting out of the house and engaging in positive activity	Daytime activities Employment and volunteering	Work	It is important to have fun I do things that are meaningful to me I continue to have new interests I do things that are valuable and helpful to others I do things that give me a feeling of great pleasure
Having a better social life	Company Intimate relationships	Social networks Relationships	It is important to have a variety of friends I have friends who have also experienced mental illness I have friends without mental illness I have friends that can depend on me I feel OK about my family situation
Improved physical health	Physical health	Physical health and self-care	It is important to have healthy habits

*Notes: CANSAS* Camberwell Assessment of Need – Short Appraisal Scale [10], *MHRS* Mental Health Recovery Star [28], *RAS-DS* Recovery Assessment Scale (Domains and Stages) [29]

In reviewing the information presented in Table 2, it is clear that none of the measures fully capture all outcomes reported by study participants. For example, the RAS-DS does not cover *improved physical health* and the items related to *improved mental clarity, focus and order in life* primarily refer to illness management and overall competence rather than specific life management skills (like keeping the house in order). On the other hand, *getting out of the house and engaging in positive activity* is only covered in terms of work in MHRS and *feeling supported* is also only partially covered. This demonstrates that multiple measures may be needed to capture the range of both recovery-focused and needs-based outcomes that consumers desire and can achieve from a service coordination and brokerage type service.

Other findings from this study are also worthy of further exploration, most notably in the areas of engagement in meaningful activities and improving social connections. The lack of meaningful activities and the social isolation faced by individuals with severe and persistent mental illness is well documented [30–34]. At intake, CANSAS items of *Daily activity* and *Company* were each rated as “unmet needs” for over 50% of PIR consumers [11]. While participants in this study identified improvements in these areas, there is evidence that further development in these domains is likely to be required. Similar findings have also been reported from other PIR studies [35]. In both domains, the outcomes discussed by participants were focused around the early stages of recovery.

While the recovery journey is non-linear, it is understood to involve a number of stages [36, 37]. For example, participants described getting out of the house and engaging in more typical daily routines, but there were few mentions of gaining and maintaining socially or personally valued roles. Similarly, participants mainly described improvements in social connectedness as a sense of social connection with their SF. While some participants described also making and sustaining connections with others, these were in the minority and there was little discussion of reciprocity in relationships.

Experiencing meaningful activities, having a variety of non-professional social connections and relationship reciprocity have all been identified as aspects of recovery that tend to be experienced more frequently by individuals who are further along in their recovery journeys [37, 38]. This may well reflect the clientele of PIR, who are people with long-term, serious mental illness and complex needs, and the relatively short-term nature of the engagement of PIR in consumers’ lives [5]. It is critical then that SFs not only provide consumers with support, but focus on facilitating consumers’ natural support systems and social connections outside the service system, to support consumers’ ongoing progress in their recovery journeys beyond the PIR service period [39]. Additionally, these further challenges suggest that a more nuanced evaluation of progress may be required in outcome measures. Using the blunt measure of “met need” versus “unmet need” provided in the CANSAS risks losing important information about progress.

### Limitations

As with most qualitative research, the applicability of the findings needs to be considered with reference to participant characteristics. Participants all came from Sydney’s North Shore and Beaches. Cultural and social differences between populations may limit applicability of findings across settings. As participation was voluntary, there is also the potential that participants who had positive experiences of their engagement with PIR services may be over-represented in the participant group. As constructivist research is unapologetically interpretive, readers should also consider the findings in relation to the positioning of the researchers themselves as described in the study methods. However, the leadership of consumers in decision-making around the research questions, the methods used to answer them and the analysis and reporting of data lends validity to the study in terms of its relevance to consumers and consumer-centred services.

## Conclusions

Notwithstanding the limitations, this research provides evidence of the outcomes that consumers value and have achieved in a care-coordination model of service. These outcomes reflect a broad range of elements that contribute to recovery [26, 27, 40]. Findings suggest that measuring change in unmet needs alone is insufficient to capture changes experienced and prioritised by individuals. Even in a service coordination model which includes no “therapeutic” intervention, a range of recovery-based outcome measures appear both appropriate and necessary to capture service outcomes that are important to consumers. Outcome measures should include measures of recovery and consumers’ perceptions of change in the six domains identified in this study.

## Abbreviations

CANSAS: Camberwell Assessment of Need – Short Appraisal Scale
CHIME: Connectedness; Hope and optimism about the future; Identity; Meaning in life; and Empowerment
MHRS: Mental Health Recovery Star
PIR: Partners in Recovery
RAS-DS: Recovery Assessment Scale – Domains and Stages
SF: Support Facilitator
